# Outcomes after microsurgical treatment of lymphedema: a systematic review and meta-analysis

**DOI:** 10.1097/JS9.0000000000000210

**Published:** 2023-04-17

**Authors:** Joachim N. Meuli, Martino Guiotto, Jolanda Elmers, Lucia Mazzolai, Pietro G. di Summa

**Affiliations:** a Department of Plastic and Hand Surgery; b Medical Library, Research and Education Department; c Angiology Division, Heart and Vessel Department, Lausanne University Hospital, University of Lausanne, Switzerland

**Keywords:** lymphedema, lymphovenous anastomosis, microsurgery, vascularized lymph node transfer

## Abstract

Microsurgical treatment options for lymphedema consist mainly of lymphovenous anastomosis (LVA) and vascularized lymph node transfers (VLNTs). There are no standard measurements of the effectiveness of these interventions and reported outcomes vary among studies.

**Methods:**

A systematic review and meta-analysis were performed based on a structured search in Embase, Medline, PubMed, Cinahl, Cochrane, and ProQuest in October 2020, with an update in February 2022. Firstly, a qualitative summary of the main reported outcomes was performed, followed by a pooled meta-analysis of the three most frequently reported outcomes using a random effects model. Randomized controlled trials, prospective cohorts, retrospective cohorts, and cross-sectional and case–control studies that documented outcomes following microsurgery in adult patients were included. Studies of other surgical treatments (liposuction, radical excision, lymphatic vessel transplantation) or without reported outcomes were excluded. The study protocol was registered on PROSPERO (International Prospective Register of Systematic Reviews) (ID: CRD42020202417). No external funding was received for this review.

**Results:**

One hundred fifty studies, including 6496 patients, were included in the systematic review. The qualitative analysis highlighted the three most frequently reported outcomes: change in circumference, change in volume, and change in the number of infectious episodes per year. The overall pooled change in excess circumference across 29 studies, including 1002 patients, was −35.6% [95% CI: −30.8 to −40.3]. The overall pooled change in excess volume across 12 studies including 587 patients was −32.7% [95% CI: −19.8 to −45.6], and the overall pooled change in the number of cutaneous infections episodes per year across 8 studies including 248 patients was −1.9 [95% CI: −1.4 to −2.3]. The vast majority of the studies included were case series and cohorts, which were intrinsically exposed to a risk of selection bias.

**Conclusion:**

The currently available evidence supports LVA and vascularized lymph node transfers as effective treatments to reduce the severity of secondary lymphedema. Standardization of staging method, outcomes measurements, and reporting is paramount in future research in order to allow comparability across studies and pooling of results.

## Introduction

Highlights

- The vast majority of published studies focus on female patients and on secondary lymphedema.
- Pooled reduction of 35% in excess circumference and 32% in excess volume after microsurgery.
- Heterogeneity persists in measurement methods; standardization is of paramount importance.

### Rationale

The term lymphedema describes any pathologic lymph fluid accumulation in the interstitial compartment of the human body, regardless of whether it is primary lymphedema caused by congenital malformation of the lymph drainage system or secondary lymphedema caused by infectious diseases or by injury to lymph vessels (traumatism, radiotherapy, surgery). Primary lymphedema is a rare disease and accounts for ∼1–10% of all lymphedema cases^1^,^2^. Secondary lymphedema is more frequent, with the leading cause in low to middle-income countries being infection by nematodes, while the main etiology in high-income countries is an injury to lymph vessels during surgical or radiological cancer treatment^1^,^3^. Classical management is based on compressive dressings and manual drainage [compressive decongestive therapy (CDT)]. For cases refractory to conservative strategies, microsurgical interventions appeared in the 1960s when several different teams described lymphovenous anastomosis (LVA) in animal models^4^, followed later by reports on humans^5^–^10^. LVA techniques were later refined and are nowadays applied worldwide. Vascularized lymph node transfers (VLNT) were first described in animals in 1979 by Shesol *et al*.^11^ and in humans in 1982 by Clodius *et al*.^12^ as pedicled flaps. This technique was later refined as free flap surgery and was widely adopted as a second line of treatment or as an adjunction to LVA.

Published literature on both techniques increased drastically in the last 10 years, describing very satisfying results. However, there is no standard measurement of the effectiveness of these interventions and reported outcomes vary significantly among studies. This lack of standardization prevented the production of good-quality evidence about the efficacy and safety of these procedures. In this systematic review of the literature, we aimed to determine how the outcomes of microsurgical treatment for lymphedema were assessed in published studies. When enough studies reported similar assessment methods, we aimed to pool the results in a meta-analysis to determine the effectiveness of microsurgical therapy. In addition, data regarding adverse events associated with microsurgery for lymphedema was collected.

## Methods

Methods of this review were determined in advance and published in a protocol on PROSPERO (ID: CRD42020202417). This systematic review followed the PRISMA (preferred reporting items for systematic reviews and meta-analyses), Supplemental Digital Content 1, http://links.lww.com/JS9/A343 and AMSTAR 2 (assessing the methodological quality of systematic reviews), Supplemental Digital Content 2, http://links.lww.com/JS9/A344 guidelines^13^,^14^. A self-performed rating according to AMSTAR 2 concluded that moderate confidence could be given in the results of this review.

### Eligibility criteria

Randomized controlled trials, prospective cohort, retrospective cohort, and cross-sectional and case-series studies that documented outcomes following microsurgery for extremities lymphedema in adult (>18 years old) patients were included. We willingly renounced a systematic review and meta-analysis restricted to randomized controlled trials only as very few have been published so far, and such an analysis was unlikely to provide a comprehensive summary of the effects of microsurgical treatment of lymphedema. Microsurgery was defined as LVA or VLNT. Several variations of the LVA technique have been described, and in order to avoid unjustified exclusion of studies, all surgical techniques were included. We tried to categorize studies as using either single LVA (sLVA) or multiple LVA (mLVA, being defined as multiples lymphatics anastomosed to a single vein). Authors are, however, not always consistent in the use of these abbreviations. Studies reporting a combined treatment using both microsurgical techniques or using the microsurgical technique in combination with a flap for soft tissue reconstruction were included. Studies comparing both microsurgical techniques were included as well. Studies reporting procedures in which patients underwent microsurgical treatment followed, secondarily, by other surgical or nonsurgical treatments were included if they reported details of the assessments before and after microsurgery.

Studies of other surgical treatments (liposuction, radical excision) or without reported outcomes were excluded. Lymphatic vessel transplantation or transfer, despite being microsurgical by definition, was excluded as it is noticeably less widespread than the two other techniques. Studies about parasitic lymphedema were excluded as well. Studies reported in languages other than English were excluded.

### Information sources

To identify articles, a systematic search strategy was designed by a medical librarian, using a combination of controlled vocabulary terms and free text terms covering the two overarching concepts of the research: lymphedema and microsurgery. The search strategy was translated for a range of databases: Embase.com, Medline Ovid SP, PubMed (not Medline), Cinahl Full Text – Ebsco, Cochrane Central Register of Controlled Trials – Wiley and ProQuest Dissertations & Theses A&I. The search strategies were peer-reviewed by a second medical librarian before the initial searches were conducted in October 2020 and repeated in February 2022. The searches were performed without limits for publication date or languages. The search equations for all the databases are provided as Supplementary material 3, Supplemental Digital Content 6, http://links.lww.com/JS9/A348. All references identified through the searches were downloaded into EndNote and duplicates were removed.

### Selection process

The resulting records were uploaded to the Rayyan QCRI software. This software was used to perform screening for inclusion by two authors who graded articles for levels of evidence during the same process. When uncertainty about inclusion was present, a supplementary evaluation and grading of evidence was performed by a senior author. All disagreements were resolved by consensus among the three reviewers.

### Data collection process

Data collection was performed by two authors under the supervision of one senior author. When needed, study investigators were contacted by e-mail to obtain or confirm data.

### Data items

In the qualitative part of the review, the assessment methods used in the included studies were retrieved and classified according to these preselected categories: volume, circumference, number of CDT per defined period of time, use of compressive garments, number of infectious episodes per defined period of time, lymphoscintigraphy, lymphography, magnetic resonance imaging/computed tomography/single-photon emission computed tomography-computed tomography (MRI/CT/SPECT-CT), quality of life (QoL) questionnaires, and patient satisfaction. In the second part of the review, quantitative results for the three most frequently reported outcomes were retrieved from all concerned studies and pooled for a meta-analysis.

In parallel to these outcomes, data relative to study location, participants’ characteristics (sex, age, BMI), etiology of lymphedema, localization of lymphedema, preoperative conservative therapy attempts, staging [International Society of Lymphology (ISL) scale or other staging scales], duration of symptoms, surgical technique used, the mean number of anastomosis (for LVA) and mean follow-up duration was retrieved as well, Supplementary material 4, Supplemental Digital Content 7, http://links.lww.com/JS9/A349.

### Study risk of bias assessment/study quality

For studies included in the meta-analysis, randomized controlled trials’ methodological quality was assessed with the Cochrane risk-of-bias tool. Cohort and case-series studies were assessed using a modified version of the Newcastle–Ottawa Scale (NOS) in which the items relating to controls and comparability were removed due to the type of studies included. This change from the protocol published on PROSPERO was decided in order to improve comparability across surgical specialties.

### Effect measures

The three most frequently reported outcomes were the change in excess circumference, the change in excess volume, and the change in the number of cutaneous infections per year (see the ‘Results’ section). The effect size used in the meta-analysis for the change in excess circumference was the reduction, in percentage, of the circumference difference between the healthy limb and the affected contralateral limb. When cohort studies reported changes for different arms without reporting the change for the overall cohort, the reported variations and standard deviations were pooled. When studies reported changes with a range instead of standard deviation, we did not input the latter in accordance with the Cochrane Handbook recommendations, and studies were excluded. When studies reported raw data without summary for the entire set of patients, mean and standard deviations were calculated from the data available. For the change in excess volume, the selected effect size was the reduction, in percentage, of the volume difference between the healthy limb and the affected contralateral limb. The same methods were used to retrieve mean values and standard deviations if they were not directly provided in the paper. The effect size for the change in the number of cutaneous infections per year was the raw number of such episodes across 12 months.

### Synthesis methods

#### Qualitative analysis

All studies were included in this analysis.

#### Quantitative analysis

Only studies that provided the adequate effect size and the necessary associated standard deviation were included in the meta-analysis for each outcome. All multi-arms designs (LVA vs. VLNT, lower vs. upper extremity; LVA-technique A vs. LVA-technique B) were treated by groups combination in order to avoid both double-counting and correlated effect sizes, according to the Cochrane recommendations^15^. We planned a subgroup analysis for each surgical technique if at least 10 studies could be included. The pooling of the effect sizes was performed using a random effects model in order to account for the considerable heterogeneity between the studies. The restricted maximum likelihood estimator^16^ was used to calculate the heterogeneity variance τ2. We used Knapp–Hartung adjustments^17^ to calculate the confidence interval around the pooled effect. An outlier and influence analysis was performed for all pooled effect sizes. All analyses were performed using the R Project for Statistical Computing version 3.6.2. A *P* value less than 0.05 was considered statistically significant.

## Results

### Study selection

**Figure 1:**
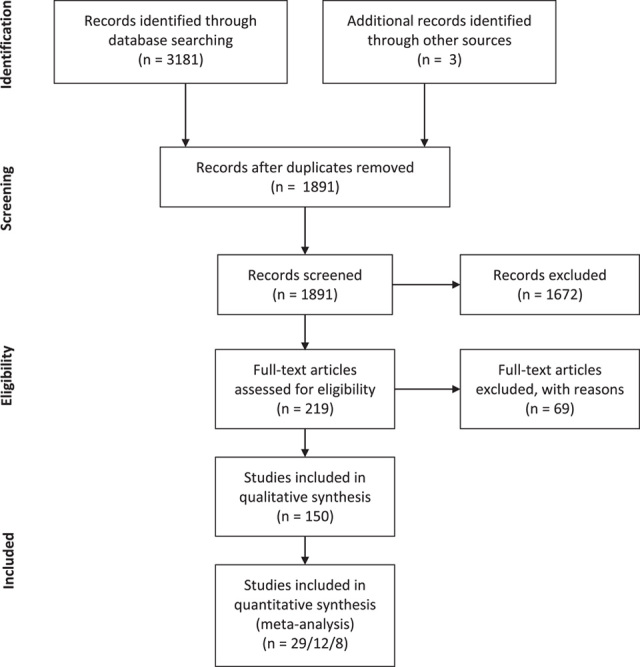
PRISMA (preferred reporting items for systematic reviews and meta-analyses) flowchart.

The original search yielded 3181 results. Three articles were identified through other sources. After the removal of duplicates, 1891 records were screened and 219 full-text articles were assessed for eligibility. One thousand six hundred seventy-two records were excluded at this stage. After the full-text assessment, another 69 articles were excluded, leaving 150 articles for the qualitative analysis^9^,^18^–^165^. Eleven articles amongst these 69 formally met the inclusion criteria but were nonetheless excluded due to insufficient reporting of patients’ characteristics, insufficient reporting of outcomes and/or overlapping of patients’ populations (Fig. 1: PRISMA flowchart). Eight of those 11 studies, ranging from 2001 to 2016, encompassed a broad population of several thousand patients and played a significant role in the popularization and refinement of LVA techniques. The absence of precise population sizes and characteristics, as well as insufficient outcomes data, however, prevented their inclusion in our systematic review and meta-analysis. A list of all excluded studies with justification for the exclusion is available as Supplementary material 2, Supplemental Digital Content 4, http://links.lww.com/JS9/A346.

The 150 included articles reported on 6496 patients and covered a period from 1990 to 2022. The number of patients included in each study ranged from 5 to 664 (mean: 43 patients). Gender was not reported for 1751 patients. However, 4335 of the remaining 4745 patients (91%) were female and 410 were male (9%). The etiology of lymphedema was frequently reported (138/151 studies): 92% of the cases were secondary lymphedema, amongst which 58% were breast carcinoma-related lymphedema. Seven percent of the cases were primary lymphedema and 1% was of unreported etiology. The localization was almost always reported (149/150 studies) with a fairly equal repartition between the lower and upper extremities: 52% of the cases concerned the lower extremity and 48% the upper extremity. Duration of symptoms and duration of follow-up were reported in 96/150 (64%) and 127/150 (85%) studies. The average symptom duration was 66.8±32.6 months. The average follow-up was 20.4±16.5 months. There were two randomized controlled trials, one randomized trial, 29 prospective and 35 retrospective cohorts, 30 prospective case series, and 53 retrospective case series. Eighty studies included only secondary lymphedema, while 54 studies included a mix of primary and secondary lymphedema cases and one study focused on primary lymphedema only. The remaining 16 studies had either no available information or incomplete information about the etiology. The staging method used was highly variable with 62 studies using the International Society of Lymphology (ISL) scale, while 43 studies reported results using Campisi, Cheng, Yamamoto, indocyanine green leg dermal backflow (ICG-LDB/DBF) or custom staging systems and 45 studies reporting no stages for the included patients. Regarding the technique, 89 studies investigated LVA, 48 studies VLNT, and 13 a combination of both procedures. Eighty-eight percent (78/89) of the studies reporting about LVA included sLVA. Four percent (4/89) included mLVA and 8% included either a combination of both or an unspecified technique. The characteristics of all included studies are described in a table available as Supplementary material 1: included studies, Supplemental Digital Content 5, http://links.lww.com/JS9/A347.

### Results of syntheses

#### Qualitative analysis

In the 150 articles included, the most frequently reported outcomes were the change in circumference (100/150 studies, 67%), the change in volume (54/150 studies, 36%), and the change in number of cutaneous infections (53/150 studies, 35%). Several studies reported more than one outcome. Changes in quality of life (QoL) questionnaire scores were reported in 22% of studies. Lymphoscintigraphic changes were reported in the same proportion as patient satisfaction (21% of studies) and changes in the use of compressive garments were reported in 19% of studies. Lastly, changes in the number of CDT sessions in lymphography and MRI/CT/SPECT-CT follow-up investigations were respectively reported in 11, 7, and 3% of all included studies.

#### Quantitative analysis

**Figure 2:**
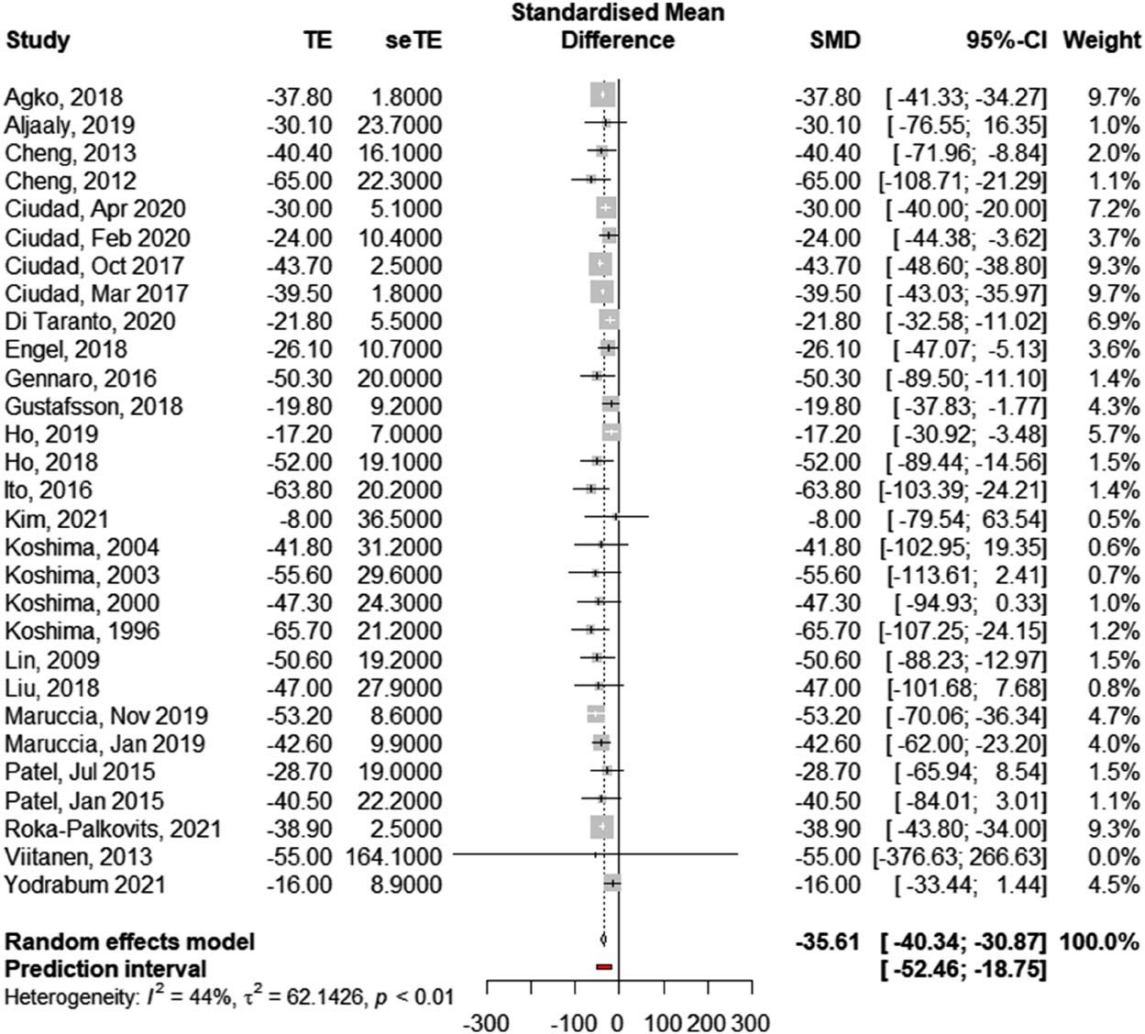
Pooled change in excess circumference after microsurgical therapy using a random effect model. Effect size  = Change in excess circumference in percentage.

*Primary outcome: change in excess circumference.* Among the 100 studies that reported changes in circumference, 42 reported a change in excess circumference, 34 reported a change in upper extremity or lower extremity lymphedema index (UEL index or LEL index), 17 reported a change in absolute circumference, and 7 reported another type of change (e.g. arbitrary categorical cutoffs). In order to be able to pool effect sizes, we limited the meta-analysis to the most frequently reported effect, that is the change (in percentage) in excess circumference (Table 1: results of individual studies). Twenty-nine studies could be included and the remaining 13 had to be excluded because necessary data were missing. Twenty out of these 29 studies investigated VLNT, 8 investigated LVA, and 1 investigated a combination of both techniques. The overall pooled change in excess circumference for the 1002 patients in these 29 studies was −35.6% [95% CI: −30.9 to −40.3] (Fig. 2: forest plot). The between-study heterogeneity variance was estimated at *τ* ^2^=62.12 [95% CI: 4.13–160.31], with an *I* ^2^ value of 44.3% [95% CI: 13.5–64.1%]. The prediction interval ranged from −18.8% to −52.4%. An influence analysis confirmed that no study differed significantly from the others and/or contributed disproportionately to heterogeneity.

**Table 1:** Change in excess circumference (%).

Author	*n*	Mean (SD)	Origin of effect size	Technique
Agko *et al*., 2018^92^	12	−37.8 (1.8)	Supplied in paper	VLNT
Aljaaly *et al*., 2019^111^	15	−30.1 (23.7)	Supplied in paper	VLNT
Cheng *et al*., 2013^45^	10	−40.4 (16.1)	Supplied in paper	VLNT
Cheng, 2012^166^	6	−65.0 (22.3)	Calculated from data	VLNT
Ciudad *et al*., Apr 2020^139^	6	−30.0 (5.1)	Supplied in paper	VLNT
Ciudad *et al*., Feb 2020^140^	83	−24.0 (10.4)	Supplied in paper	VLNT
Ciudad *et al*., Oct 2017^72^	7	−43.7 (2.5)	Supplied in paper	VLNT
Ciudad *et al*., Mar 2017^71^	10	−39.5 (1.8)	Supplied in paper	VLNT
Di Taranto *et al*., 2020^122^	10	−21.8 (5.5)	Calculated from subgroups	VLNT
Engel *et al*., 2018^89^	72	−26.1 (10.7)	Calculated from subgroups	VLNT+LVA
Gennaro *et al*., 2016^68^	69	−50.3 (20.0)	Calculated from data	LVA
Gustafsson *et al*., 2018^85^	35	−19.8 (9.2)	Supplied in paper	VLNT
Ho *et al*., 2019^106^	76	−17.2 (7.0)	Supplied in paper	VLNT
Ho *et al*., 2018^86^	43	−52.0 (19.1)	Supplied in paper	VLNT
Ito *et al*., 2016^65^	5	−63.8 (20.2)	Supplied in paper	LVA
Kim *et al*., 2021^157^	133	−8.0 (36.5)	Supplied in paper	LVA
Koshima *et al*., 2004^23^	52	−41.8 (31.2)	Supplied in paper	LVA
Koshima *et al*., 2003^22^	13	−55.6 (29.6)	Supplied in paper	LVA
Koshima *et al*., 2000^20^	12	−47.3 ( 24.3)	Calculated from data	LVA
Koshima *et al*., 1996^19^	6	−65.7 (21.2)	Supplied in paper	LVA
Lin *et al*., 2009^26^	13	−50.6 (19.2)	Supplied in paper	VLNT
Liu *et al*., 2018^94^	30	−47.0 (27.9)	Supplied in paper	VLNT
Maruccia *et al*., Nov 2019^103^	16	−53.2 (8.6)	Calculated from subgroups	VLNT
Maruccia *et al*., Jan 2019^104^	21	−42.6 (9.9)	Calculated from subgroups	VLNT
Patel *et al*., Jul 2015^47^	25	−28.7 (19.0)	Calculated from subgroups	VLNT
Patel, Jan 2015^48^	20	−40.5 (22.2)	Supplied in paper	VLNT
Roka-Palkovits *et al*., 2021^153^	70	−38.9 (2.5)	Supplied in paper	VLNT
Viitanen *et al*., 2013^44^	14	−55.0 (164.1)	Calculated from data	VLNT
Yodrabum *et al*., 2021^148^	118	−16.0 (8.9)	Calculated from subgroups	LVA

LVA, lymphovenous anastomosis; VLNT. vascularized lymph node transfers.

**Figure 3:**
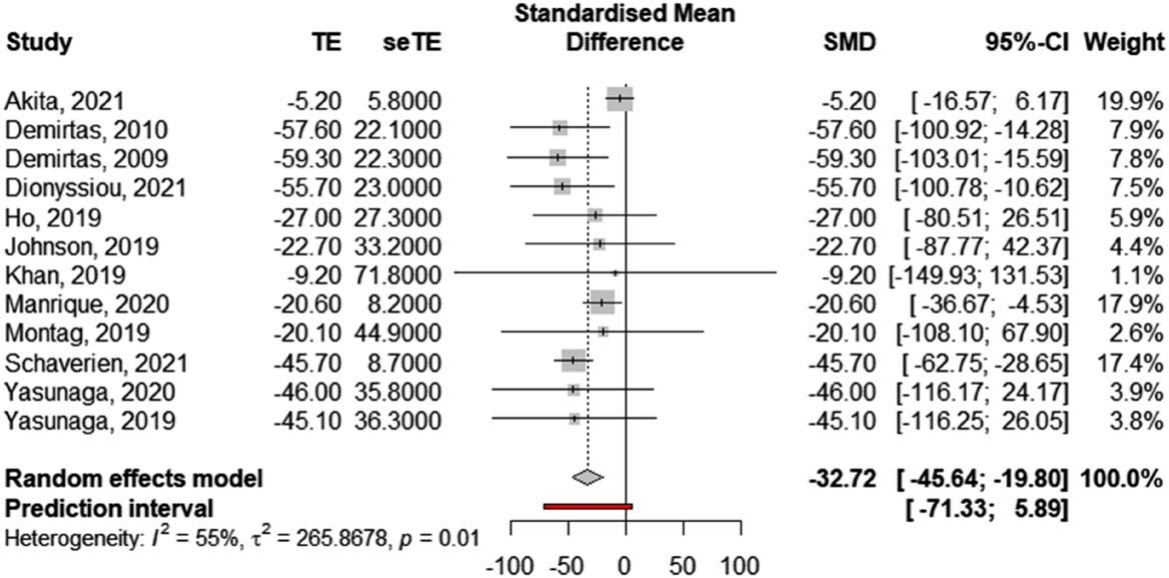
Pooled change in excess volume after microsurgical therapy using a random effect model. Effect size  = Change in excess volume in percentage.

*Secondary outcomes: change in excess volume and change in cutaneous infections.* Data regarding volume variation was available in 54 out of 150 studies. Among these 54 studies, 39 reported a change in excess volume, 7 reported a change in absolute volume, and 8 studies reported another type of volume change. As for the primary outcome, we limited the meta-analysis to studies reporting the same effect size, namely the change, in percentage, in excess volume (Table 2: results of individual studies and Fig. 3: forest plot). Twelve studies provided sufficient data to be included. Five of these 12 studies investigated LVA, 6 investigated VLNT, and 1 investigated a combination of both techniques. The overall pooled change in excess volume for the 587 patients included in these studies was −32.7% [95% CI: −19.8 to −45.6] (Fig. 3: forest plot). The between-study heterogeneity variance was estimated at *τ* ^2^=265.86 [95% CI: 7.07–579.05], with an *I* ^2^ value of 54.7% [95% CI: 13.2–76.4%]. The prediction interval ranged from +5.9% to −71.3%. Akita *et al*.^145^ were an outlier with a significantly smaller change in excess circumference with a more limited standard deviation. When excluded, the overall pooled change in excess volume of the remaining 11 studies increased slightly to −38.4% [95% CI: −27.7 to −49.1], with a notable reduction in heterogeneity (*I* ^2^=0.0%, 95% CI: 0.0–60.2%) and in prediction interval (−15.5% to −63.2%).

**Table 2:** Change in excess volume (%).

Author	*n*	Mean (SD)	Origin of effect size	Method	Technique
Akita *et al*., 2021^145^	60	−5.2 (5.8)	SD calculated from subgroups	Truncated cone formula	VLNT/LVA
Demirtas *et al*., 2010^29^	78	−57.6 (22.1)	Supplied in paper	Software calculation on serial measurements	LVA
Demirtas *et al*., 2009^28^	42	−59.3 (22.3)	Supplied in paper	Software calculation on serial measurements	LVA
Dionyssiou *et al*., 2021^159^	64	−55.7 (23.0)	Supplied in paper	Truncated cone formula	VLNT
Ho *et al*., 2019^106^	76	−27.0 (27.3)	SD calculated from subgroups	CT scan	VLNT
Johnson *et al*., 2019^107^	7	−22.7 (33.2)	SD calculated from data	Not reported	VLNT
Khan *et al*., 2019^98^	27	−9.2 (71.8)	Supplied in paper	Perometry	LVA
Manrique *et al*., 2020^130^	26	−20.6 (8.2)	SD calculated from subgroups	Truncated cone formula	VLNT
Montag *et al*., 2019^117^	24	−20.1 (44.9)	Supplied in paper	Truncated cone formula	VLNT
Schaverien *et al*., 2021^152^	134	−45.7 (8.7)	Supplied in paper	Perometry	VLNT
Yasunaga *et al*., 2020^126^	19	−46.0 (35.8)	Supplied in paper	Bioimpedance	LVA
Yasunaga *et al*., 2019^112^	30	−45.1 (36.3)	Supplied in paper	Bioimpedance	LVA

CT, computed tomography, LVA, lymphovenous anastomosis; VLNT. vascularized lymph node transfers.

**Figure 4:**
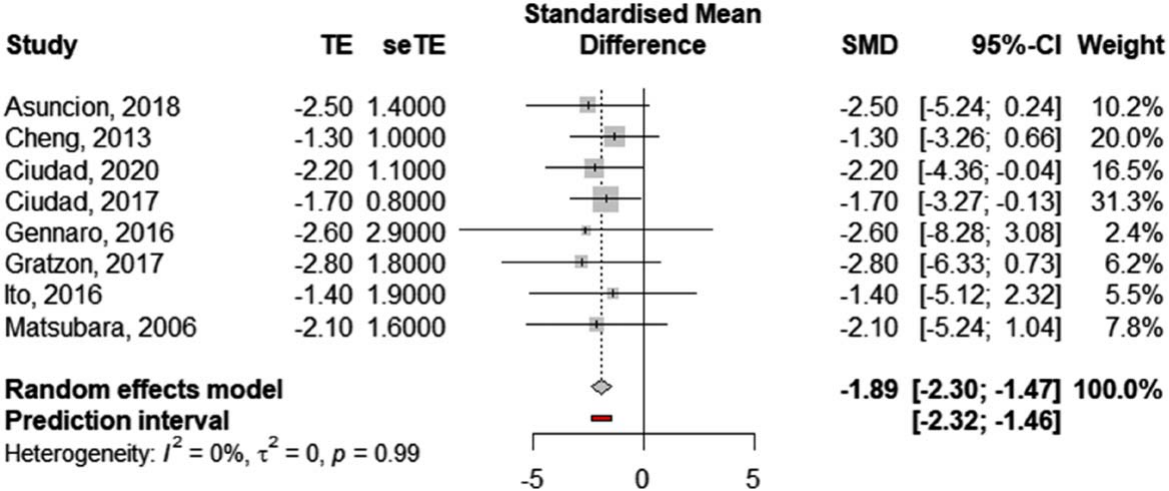
Pooled change in cutaneous infections using a random effect model. Effect size  = Change in number of cutaneous infections per year (absolute value).

A change in cutaneous infections was reported in 48 out of 151 studies: 38 reported a change in the number of infectious episodes per year, 8 studies a change in the number of patients affected per year, and 2 used another form of a report. Eight out of the 40 studies provided sufficient data to be included in the meta-analysis (Table 3: results of individual studies and Fig. 4: forest plot). Five out of these eight studies investigated VLNT and three investigated LVA. The overall pooled change in the number of cutaneous infections episode per year for the 248 patients included was −1.9 [95% CI: −1.4 to −2.3] (Fig. 3: forest plot). The between-study heterogeneity variance was estimated at *τ* ^2^=0, with an *I* ^2^ value of 0.0% [95% CI: 0.0–67.6%]. The prediction interval ranged from −1.5 to −2.3. The heterogeneity and influence analysis did not highlight any significant outlier.

**Table 3:** Change in number of infectious episodes per year (n).

Author	*n*	Mean (SD)	Origin of effect size	Technique
Asuncion *et al*., 2018^88^	15	−2.5 (1.4)	SD calculated from data	VLNT
Cheng *et al*., 2013^45^	10	−1.3 (1.0)	Supplied in paper	VLNT
Ciudad, 2020^140^	83	−2.2 (1.1)	Supplied in paper	VLNT
Ciudad, 2017^72^	7	−1.7 (0.8)	SD calculated from data	VLNT
Gennaro *et al*., 2016^68^	69	−2.6 (2.9)	SD calculated from data	LVA
Gratzon *et al*., 2017^76^	50	−2.8 (1.8)	SD calculated from data	VLNT
Ito *et al*., 2016^65^	5	−1.4 (1.9)	Supplied in paper	LVA
Matsubara *et al*., 2006^24^	9	−2.1 (1.6)	SD calculated from data	LVA

LVA, lymphovenous anastomosis; VLNT. vascularized lymph node transfers.

## Discussion

The systematic review highlighted a certain homogeneity in the patients’ populations with a large majority of cases involving female patients and lymphedema of secondary etiology. Several key demographics were, however, frequently missing: the gender distribution was not specified for a quarter of the population and characteristics such as age and BMI were reported with their standard deviations in less than half of the studies. In addition, a large variety of staging systems was applied by the different authors (International Society of Lymphology, Campisi’s, Cheng’s, ICG-LDB). We observed a high heterogeneity between the studies in terms of measurement units, measurement methods as well as grouping distributions. For the most frequently reported outcome (changes in volume), some authors reported changes in excess volume, and some others reported a change in the absolute volume of the limb. Results were reported using several different techniques: serial circumferential measurements and calculations, perometry, water volumetry, CT scan, MRI, or bioimpedance. Variations in circumferences were as well reported, sometimes as changes in excess and sometimes as changes in absolute circumference. The measurement sites on the extremity were not standardized, and some authors preferred to report changes in percentages, in raw centimetric value, or using the LEL and UEL indexes. We observed the same variability on data assessment for the other outcomes such as frequency of infectious episodes or CDT sessions. This heterogeneity was expected but nonetheless limited the number of studies that could eventually be included in the meta-analysis. Another example of such variability is found in the reporting of quality of life changes. The majority of studies (19/33) reported such changes using the LYMQOL scale or one of its components but some other scales were frequently used as well (Lymph-ICF, LeQOLIS, LLIS, and custom scales). Amongst studies using the LYMQOL scale, none reported values with standard deviation and a meta-analysis was therefore not possible. This is unfortunate given the dramatic variations reported by some authors: the 14 studies that included the overall QoL score of LYMQOL reported increases ranging from 16 to 240% with a preoperative score ranging from 2.2 to 5.8 and a postoperative score ranging from 6.3 to 8.2.

It is worth noting that the scoring system described in the original article from Keeley *et al*.^167^ does not provide a recommended way of summarizing results. As a consequence, some authors summed the scores of all questions while others reported only the score of the ‘overall QoL’ question. The fact that, in the first case, a higher score reflects a lower quality of life while it is the opposite in the second case only adds to the confusion. We therefore strongly advocate for future studies to use only the overall QoL score of the LYMQOL scale unless these studies are specifically focused on quality of life outcomes.

Our reported pooled reduction of 35% in excess circumference following microsurgical treatment for lymphedema is a promising result. The pooled reduction in excess volume of 33% and the significant reduction in the number of cutaneous infections per year are exciting results as well.

A similar systematic review and meta-analysis were recently published without previous publication of the protocol^168^. It included 66 studies with similar outcomes, despite the application of broader inclusion criteria (notably, the inclusion of liposuction therapies) and slightly different effect sizes. Thirty-nine of the 66 studies (59%) included in this review were included in our systematic review as well. The remaining 27 studies were excluded from our selection because they either reported results after liposuction (14 studies), reported the effects of preventive microsurgical therapy (6 studies), included cases of filariasis (2 studies), included pediatric cases (1 study), reported the treatment of lymphocele (1 study), were published in a language other than English and/or could not be accessed (3 studies). The pooled effect size of our meta-analysis cannot be directly compared with Chang *et al*.’s, but our findings are overall coherent with this previous work which reported a pooled reduction of 3.80 [95% CI: 2.93–4.67] cm in circumference after treatment with LVA and 1.64 cm [95% CI: 0.87–2.42] after treatment with VLNT.

These results should be approached with precaution as the validity of our meta-analysis is entirely based on the validity of the included studies. Only two of these were randomized controlled trials that can guarantee a superior level of evidence. The vast majority of the studies included in the systematic review and all studies included in the meta-analysis were nonrandomized studies of interventions (NRSI) which intrinsically exposed to a risk of selection bias. As recently demonstrated by the RE-ENERGIZE Trial, the pooling of results in a meta-analysis of NRSI may result in a statistically significant estimate of an effect of great magnitude that is however not reproducible in a randomized, placebo-controlled trial^169^,^170^. Another issue is the pressure to publish positive results that might have precluded the publication of nonsignificant changes after surgery or, when published, might have precluded inclusion in the meta-analysis because necessary data were not reported^27^,^155^.

In spite of these limitations, this systematic review’s validity is supported by its extensive reach. It joins a growing set of literature^171^–^175^ that suggests LVA and vascularized lymph node transfers are effective treatments to reduce the severity of secondary lymphedema of the extremities.

The standardization of staging, outcomes measurements, and their reporting is a paramount next step in order to allow comparability between studies and the pooling of results. The most objective, reproducible, and standardized outcome available at the moment is the Kleinhans transport index calculated on lymphoscintigraphy^176^. The standardization of the procedure and publication of ‘standard protocols’ are recent achievements^177^,^178^ and offer researchers the opportunity to compare studies across centers and countries. Large randomized and case–control trials are underway for both surgical techniques (NCT03578380 at Sykehuset Telemark in Norway^179^, NCT03941756 at MD Anderson Cancer Center in the USA^180^, and NCT05064176 at Universitaire Ziekenhuizen Leuven in Belgium^181^ for LVA; NCT03248310 at Memorial Sloan Kettering Cancer Center in the USA^182^ for VLNT) and should provide further data between 2023 and 2025.

Hopefully, the results of these trials will provide high-quality evidence that can be translated into clinical-practice recommendations to guide healthcare providers in patient selection and in the choice of the adequate treatment.

## Ethical approval

No ethical approval is necessary (systematic review and meta-analysis).

## Sources of funding

The authors did not receive any funding for this study.

## Author contribution

J.M., J.E., and P.D.S.: designed the study; J.M., J.E., and M.G.: collected the data; J.M.: analyzed the data; J.M., J.E., M.G., L.M., and P.D.S.: wrote the manuscript. All authors contributed and approved the final version of the manuscript.

## Research registration unique identifying number (UIN)

1. Name of the registry: PROSPERO.
2. Unique identifying number or registration ID: CRD42020202417.
3. Hyperlink to your specific registration (must be publicly accessible and will be checked): https://www.crd.york.ac.uk/prospero/display_record.php?RecordID=202417

## Guarantor

Prof Pietro Di Summa, MD, PhD, Plastic and Hand Surgery Department, Lausanne University Hospital.

## Conflicts of interest disclosure

The authors do not report any conflicts of interest.

## Data statement

How effective is lymphatic microsurgery? A systematic review and meta-analysis of outcomes after microsurgical treatment of lymphedema

## Research data for this article

Data extracted from included studies and data used for all analyses are available from the corresponding authors upon reasonable request. Analytic code is freely available in the R Project for Statistical Computing.

## Provenance and peer review

Not commissioned, externally peer-reviewed.

## Supplementary Material
