# Supplementary material for: Outcomes after microsurgical treatment of lymphedema: a systematic review and meta-analysis
Source: Int J Surg. 2023 Apr 17;109(5):1360–72. doi: 10.1097/JS9.0000000000000210 (PMC10389392; doi:10.1097/JS9.0000000000000210)
Supplement: Supplementary file 3 [file js9-109-1360-s003.pdf]

Author	Year	Title	Reason for exclusion
Bolletta, A	2022	Combined lymph node transfer and suction-assisted lipectomy in lymphedema treatment: A prospective study	Results reported only for combination of GEVLTN and liposuction
Scaglioni, MF	2022	Combining superficial and deep lymphovenous anastomosis for lymphedema treatment: Preliminary results	No outcomes data reported
Bamba, R	2021	Outcomes analysis of microsurgical physiologic lymphatic procedures for the upper extremity from the United States National Surgical Quality Improvement Program	No outcomes data reported
Bianchi, A	2021	Recipient Venule Selection and Anastomosis Configuration for Lymphaticovenular Anastomosis in Extremity Lymphedema: Algorithm Based on 1,000 Lymphaticovenular Anastomosis	No outcomes data reported
Chamberlain, K et al.	2021	Quality of Life Outcomes Based on Recipient Site Choice in Vascularized Lymph Node Transfer for Treatment of Breast Cancer-Related Lymphedema	Systematic review and protocol for further study
Chang, D. W.	2021	Combined Approach to Surgical Treatment of Lymphedema	No outcomes data reported
Ciudad, P. et al.	2021	A single-stage triple-inset vascularized gastroepiploic lymph node transfers for the surgical treatment of extremity lymphedema	Letter to the editor
Ciudad, P. et al.	2021	Preoperative indocyanine green lymphographic planning for dorsal metatarsal vascularized lymph vessel transfer in the treatment of upper extremity lymphedema	Letter to the editor
Pons, G. et al.	2021	Reverse Lymph Node Mapping Using Indocyanine Green Lymphography: A Step Forward in Minimizing Donor-Site Morbidity in Vascularized Lymph Node Transfer	No outcomes data reported
Sakai, H.	2021	Evidence of lymph flow amelioration on indocyanine green lymphography after vascularized lymph node transfer	Letter to the editor
Campisi C	2021	Matching primary with secondary lymphedemas across lymphatic surgery in genoa (italy) from 1973 until time of covid-19	Incomplete outcomes data prevented inclusion
Cheng, M. H. and Tee, I	2020	Simultaneous Ipsilateral Vascularized Lymph Node Transplantation and Contralateral Lymphovenous Anastomosis in Bilateral Extremity Lymphedema with Different Severities	Included pediatric patients
Ciudad, P. and Forte, A	2020	Impact of body mass index on long-term surgical outcomes of vascularized lymph node transfer in lymphedema patients	Same set of patients than a more recent study of the same author
Koide, S. and Lin, C. Y. a	2020	Delayed primary retention suture for inset of vascularized submental lymph node flap for lower extremity lymphedema	No outcomes data reported; same set of patients than another study of the same author
Pereira, N. and Cambar	2020	Prevention and Treatment of Posttraumatic Lymphedema by Soft Tissue Reconstruction With Lymphatic Vessels Free Flap: An Observational Study	Other surgical technique
Chen, W. F. and Knacks	2020	Delayed Distally Based Prophylactic Lymphaticovenular Anastomosis: Improved Functionality, Feasibility, and Oncologic Safety?	Letter to the editor
van Mulken TJM	2020	First-in-human robotic supermicrosurgery using a dedicated microsurgical robot for treating breast cancer-related lymphedema: a randomized pilot trial	Same set of patients than a more recent study of the same author
Suzuki, Y. and Sakuma,	2019	Evaluation of patency rates of different lymphaticovenous anastomosis techniques and risk factors for obstruction in secondary upper extremity lymphedema	Unsufficient/missing population characteristics
Yoshida, S. and Koshim	2019	Line production system for multiple lymphaticovenular anastomoses	Unsufficient/missing population characteristics
Kraft, C. T. and Eiferma	2019	Complications after vascularized jejunal mesenteric lymph node transfer: A 3-year experience	No outcomes data reported
Brebant, V. and Heine,	2019	Augmented reality of indocyanine green fluorescence in simplified lymphovenous anastomosis in lymphatic surgery	Unsufficient/missing population characteristics
Yoshida, S. and Koshim	2019	Mechanical Dilation Using Nylon Monofilament Aids Multisite Lymphaticovenous Anastomosis Through Improving the Quality of Anastomosis	Unsufficient/missing population characteristics
Koshima, I. and Yoshida	2019	Effect of pregnancy on lower limb lymphedema in patients treated with multisite lymphaticovenular anastomoses (MLVAS)	No outcomes data reported
Atta, A. T.	2019	Mrl and clinical evaluation of free lymph node transfer in management of lymphoedema of extremities	Congress report
Campisi, C.	2019	Single-site multiple lymphatic venous anastomosis (SS-MLVA) for the staging-guided peripheral lymphedematreatment and consistent long-term clinical outcomes	Congress report
Cheng, M. H. and Loh, (	2018	Outcomes of Vascularized Lymph Node Transfer and Lymphovenous Anastomosis for Treatment of Primary Lymphedema	Unsufficient/missing population characteristics
Cheng MH	2018	Validity of the Novel Taiwan Lymphoscintigraphy Staging and Correlation of Cheng Lymphedema Grading for Unilateral Extremity Lymphedema	Unsufficient/missing population characteristics
Akita, S. and Ogata, F. a	2017	Noninvasive screening test for detecting early stage lymphedema using follow-up computed tomography imaging after cancer treatment and results of treatment with lymphaticovenular anastomosis	Unsufficient/missing population characteristics
Batista, B. N. and Germ	2017	Lymph node flap transfer for patients with secondary lower limb lymphedema	Included pediatric patients
Cheng, M. H. and Lin, C	2017	A prospective clinical assessment of anatomic variability of the submental vascularized lymph node flap	No outcomes data reported
Chiewvit, S. and Kumne	2017	Lymphoscintigraphic Findings That Predict Favorable Outcome after Lymphaticovenous Anastomosis	Included pediatric patients
Lee, K. T. and Park, J. W	2017	Serial two-year follow-up after lymphaticovenular anastomosis for the treatment of lymphedema	Included pediatric patients
Maldonado, A. A. and C	2017	The use of supraclavicular free flap with vascularized lymph node transfer for treatment of lymphedema: A prospective study of 100 consecutive cases	No outcomes data reported
Maegawa, J. and Matsu	2017	Mid- and long-term results of patency in side-toend lymphatic venous anastomosis for treatment of upper limb lymphedema	Document could not be retrieved
Noda, Y. and Iimura, T.	2017	Analysis of the outcome of lymphovenous anastomosis using lymphoscintigraphy and spect-ct-new classification of lower-limb lymphedema	Document could not be retrieved
Ciudad P	2017	Comparison of long-term clinical outcomes among different vascularized lymph node transfers: 6-year experience of a single center's approach to the treatment of lymphedema	Same set of patients than a more recent study of the same author
Koshima, I. and Narush	2016	Lymphadiposal Flaps and Lymphaticovenular Anastomoses for Severe Leg Edema: Functional Reconstruction for Lymph Drainage System	Other surgical technique
Masia, J. and Pons, G. a	2016	Barcelona Lymphedema Algorithm for Surgical Treatment in Breast Cancer-Related Lymphedema	Same set of patients than a more recent study of the same author
Yamamoto, T. and Yosh	2016	Complete lymph flow reconstruction: A free vascularized lymph node true perforator flap transfer with efferent lymphaticolymphatic anastomosis	Case report
Campisi C	2016	A Single-Site Technique of Multiple Lymphatic-Venous Anastomoses for the Treatment of Peripheral Lymphedema: Long-Term Clinical Outcome	Unsufficient/missing population characteristics
Hara, H. and Mihara, M	2015	Indication of Lymphaticovenous Anastomosis for Lower Limb Primary Lymphedema	Included pediatric patients
Travis, E. C. and Shugg,	2015	Lymph node grafting in the treatment of upper limb lymphoedema: a clinical trial	Other surgical technique
Akita, S. and Mitsukawa	2014	Suitable therapy options for sub-clinical and early-stage lymphoedema patients	Unsufficient/missing population characteristics
Boccardo F	2013	Lymphatic microsurgery to treat lymphedema: techniques and indications for better results	Unsufficient/missing population characteristics
Olszewski WL	2013	Lymphovenous microsurgical shunts in treatment of lymphedema of lower limbs: a 45-year experience of one surgeon/one center	Unclear reporting of data
Felmerer, G. and Sattle	2012	Treatment of various secondary lymphedemas by microsurgical lymph vessel transplantation	Other surgical technique
Campisi C	2010	Microsurgery for lymphedema: clinical research and long-term results	Unsufficient/missing population characteristics
Becker, C. and Pham, D	2008	Postmastectomy neuropathic pain: results of microsurgical lymph nodes transplantation	No outcomes data reported
Campisi C	2007	Microsurgery for treatment of peripheral lymphedema: long-term outcome and future perspectives	Unsufficient/missing population characteristics
Campisi C	2006	Lymphatic microsurgery for the treatment of lymphedema	Unsufficient/missing population characteristics
Campisi C	2004	Microsurgical techniques for lymphedema treatment: derivative lymphatic-venous microsurgery	Unsufficient/missing population characteristics
Campisi C	2003	Peripheral lymphedema: new advances in microsurgical treatment and long-term outcome	Unsufficient/missing population characteristics
Campisi C	2003	Current use of microsurgery in lymphedema	Unsufficient/missing population characteristics
Campisi, C. and Boccard	2001	The use of vein grafts in the treatment of peripheral lymphedemas: long-term results	Other surgical technique
Campisi C	2001	Long-term results after lymphatic-venous anastomoses for the treatment of obstructive lymphedema	Unsufficient/missing population characteristics
Campisi, C. and Boccard	1999	Role of microsurgery in the management of lymphoedema	Document could not be retrieved
Campisi, C. and Boccard	1999	Microsurgical treatment of peripheral lymphedema: An overview upon 25 years clinical experience	Document could not be retrieved
Campisi C.	1995	Reconstructive microsurgery of lymph vessels: The personal method of lymphatic-venous-lymphatic (LVL) interpositioned grafted shunt	Other surgical technique
Egorov, Y. S. and Abalm	1994	Autotransplantation of the greater omentum in the treatment of chronic lymphedema	Included pediatric patients
Haddad, M. and Matz,	1988	Surgical treatment of limb lymphedema	Document could not be retrieved
Ipsen, T. and Pless, J. ar	1988	Experience with microlymphaticovenous anastomoses for congenital and acquired lymphoedema	Document could not be retrieved
Huang GK et al	1985	Microlymphaticovenous anastomosis in the treatment of lower limb obstructive lymphedema: analysis of 91 cases	Included filariasis patients
Krylov, V. S. and Milanc	1985	Reconstructive microsurgery in treatment of lymphoedema in extremities	Document could not be retrieved
Degni, M.	1984	Surgical management of selected patients with lymphedema of the extremities	Document could not be retrieved
Krylov, V. and Milanov,	1982	Microlymphatic surgery of secondary lymphoedema of the upper limb	Document could not be retrieved
Huang GK et al	1981	Microlymphaticovenous anastomosis for treating lymphedema of the extremities and external genitalia	Included filariasis patients
Fox, U. and Montorsi, M	1981	Microsurgical treatment of lymphedemas of the limbs	Document could not be retrieved
Fox, U. and Montorsi, M	1980	Surgical treatment of lymphedema of the limbs with lymphaticovenous anastomosis	Document could not be retrieved
Degni, M.	1974	New technique of lymphatic venous anastomosis for the treatment of lymphedema	Document could not be retrieved
