# Supplementary material for: Outcomes after microsurgical treatment of lymphedema: a systematic review and meta-analysis
Source: Int J Surg. 2023 Apr 17;109(5):1360–72. doi: 10.1097/JS9.0000000000000210 (PMC10389392; doi:10.1097/JS9.0000000000000210)
Supplement: Supplementary file 4 [file js9-109-1360-s004.pdf]

Authors	Study type	Nb patients	Etiology primary	Etiology secondary	Lower extremity	Upper extremity	Staging	Duration sympt (Mo)	Intervention	Donor site VLNT	Follow-up (Mo)	NOS score or Cochrane risk of bias
Moon et al, 2022	Retrospective cohort	71	0%	95%	48%	52%	ISL stage II-III	90,0	VLNT	lateral thoracic/inguinal	18,1	5/5
Van Mulken et al, 2022	Randomized controlled trial	20	0%	100%	0%	100%	Not specified	NR	sLVA	NA	12,0	High risk
Yang et al, 2022	Prospective cohort	141	0%	78%	100%	0%	ISL stage 0-III	44,4	sLVA	NA	26,2	5/5
Yasunaga et al, 2022	Retrospective cohort	46	0%	100%	100%	0%	ISL stage II	NR	sLVA	NA	6,0	4/5
Schiltz et al, 2022	Retrospective cohort	42	NR	NR	100%	0%	Yamamoto stages not reported	72,0	sLVA	NA	NR	2/5
Akita et al, 2021	Prospective cohort	60	7%	93%	100%	0%	ICG LDB stage 2- 5	>6	VLNT/sLVA	lateral thoracic/submental	NA	3/5
Brahma et al, 2021	Retrospective cohort	70	0%	100%	0%	100%	ISL stage I-II	NR	sLVA	NA	7,4	4/5
Cha et al, 2021	Retrospective case serie	42	12%	88%	100%	0%	ICG LDB stage 3 - 5	105,6	sLVA	NA	18,3	4/4
Chung et al, 2021	Retrospective case serie	12	0%	100%	100%	0%	ISL stage II-III	61,2	VLNT/sLVA	supraclavicular	12,9	4/4
Dionyssiou et al, 2021	Retrospective cohort	64	0%	100%	0%	100%	ISL stage I-III	NR	VLNT	inguinal	36,0	5/5
Drobot et al, 2021	Retrospective cohort	70	31%	69%	56%	44%	ISL stage (mean 2.3)	64,9	sLVA	NA	8,0	4/5
Hara et al 2021	Retrospective case serie	34	12%	88%	100%	0%	ISL stage I-III	90,0	sLVA	NA	8,6	3/4
Kim et al, 2021	Retrospective case serie	133	2%	100%	52%	48%	Not specified	46,4	sLVA	NA	11,6	3/4
Kwon et al, 2021	Retrospective case serie	17	29%	71%	100%	0%	ISL stage II-III	NR	sLVA	NA	NR	2/4
Onoda et al, 2021	Retrospective cohort	28	7%	93%	75%	25%	ISL stage II - III	NR	sLVA	NA	6,0	4/5
Rannikko et al, 2021	Retrospective case serie	67	0%	100%	0%	100%	Not specified	42,0	VLNT	inguinal	70,0	3/4
Roka-Palkovits et al, 2021	Retrospective cohort	70	10%	90%	57%	43%	Cheng's lymphedema grade (mean 2.7)	32,3	VLNT	submental	55,5	4/5
Schaverien et al, 2021	Retrospective cohort	134	3%	95%	14%	86%	MDACC stage I-IV	NR	VLNT	omentum/mesenteric/inguinal/lateral thoracic/submental	20,1	5/5
Sutherland et al, 2021	Retrospective case serie	9	0%	100%	0%	100%	ISL stage 0-II	21,0	sLVA	NA	22,0	3/4
Yang et al, 2021	Retrospective cohort	8	0%	100%	100%	0%	ISL stage 0-III	126,0	sLVA	NA	18,0	4/5
Yodrabum et al, 2021	Retrospective case serie	118	2%	98%	0%	100%	Campisi stage I-IV	87,6	sLVA	NA	8,0	3/4
Yoshida et al, Oct. 2021	Retrospective cohort	86	NR	NR	100%	0%	ISL stage I-III	94,8	sLVA	NA	19,2	5/5
Yoshida et al, Sept. 2021	Randomized trial	50	0%	100%	100%	0%	ISL stage I-III	NR	sLVA	NA	6,0	High risk
Bianchi et al, 2020	Prospective case serie	26	0%	100%	46%	54%	Not specified	NR	LVA (not specified)	NA	9,0	4/4
Ciudad et al, Feb 2020	Retrospective cohort	83	13%	87%	64%	36%	ISL stage II - III	42,0	VLNT	inguinal/supraclavicular/omentum/appendix/ilio-caecal	32,8	5/5
Ciudad et al, Apr 2020	Retrospective case serie	6	0%	100%	0%	100%	Not specified	18,6	VLNT	omentum	12,8	4/4
Chang et al, 2020	Prospective cohort	54	0%	100%	0%	100%	Not specified	NR	VLNT/sLVA	inguinal	21,5	5/5
Akita et al, 2020	Retrospective cohort	106	NR	NR	100%	0%	Not specified	NR	sLVA	NA	12,0	5/5
Beederman et al, 2020	Prospective case serie	274	NR	NR	28%	72%	ISL stage (mean 1.7)	80,4	VLNT/sLVA	supraclavicular	15,0	4/4
Tzou et al, 2020	Retrospective case serie	51	31%	69%	82%	18%	ISL stage I - III	NR	sLVA	NA	18,0	3/4
Mousavi et al, 2020	Retrospective case serie	24	0%	100%	0%	100%	Not specified	NR	VLNT	omentum	12,0	3/4
Ngo et al, 2020	Retrospective case serie	10	0%	100%	0%	100%	ISL stage I - II	33,0	VLNT	inguinal/supraclavicular	46,0	4/4
Koide et al, 2020	Retrospective case serie	70	17%	83%	100%	0%	Cheng's stage (mean 1.6)	92,9	VLNT	submental	32,0	4/4
Kristiansen et al, 2020	Prospective case serie	12	NR	NR	100%	0%	Not specified	48,0	sLVA	NA	12,0	3/4
Manrique et al, 2020	Retrospective cohort	26	12%	88%	46%	54%	ISL stage II	NR	VLNT	omentum	8,3	4/5
Wolfs et al, 2020	Retrospective case serie	25	0%	100%	0%	100%	ISL stage I - II	74,4	sLVA	NA	14,8	4/4
Qiu et al, 2020	Prospective cohort	100	6%	94%	15%	85%	ISL stage I - III	NR	sLVA	NA	25,0	5/5
Yang et al, 2020	Prospective cohort	100	NR	80%	100%	0%	ISL stage 0 - III	57,6	sLVA	NA	NR	4/5
Yasunaga et al, 2020	Retrospective case serie	19	0%	100%	0%	100%	ISL stage I - II	NR	sLVA	NA	20,9	4/4
Yoshida et al, Jul 2020	Prospective cohort	74	NR	NR	100%	0%	ISL stage I - III	73,2	sLVA	NA	NR	4/5
Yoshida et al, May 2020	Prospective cohort	113	30%	70%	100%	0%	ISL stage II - III	76,6	sLVA	NA	20,6	5/5
Lin et al, 2020	Prospective cohort	100	0%	100%	0	100%	Not specified	6,6	VLNT	inguinal/submental	6,0	4/5
Di Taranto et al, 2020	Prospective case serie	10	0%	100%	100%	0%	ISL stage II - III	NR	VLNT	omentum	24,1	3/4
Scaglioni et al, 2019	Prospective case serie	5	60%	40%	100%	0%	ISL stage II - III	NR	sLVA	NA	NR	3/4
Klingelhoefer et al, 2019	Retrospective case serie	26	0%	100%	81%	19%	ISL stage I - III	100	sLVA	NA	23,0	3/4
Gentileschi et al, 2019	Prospective case serie	12	0%	100%	100%	0%	ISL stage III	14	sLVA + ELVA	NA	12,0	3/4
Dionyssiou et al, 2019	Retrospective case serie	41	0%	100%	0	100%	ISL stage I - III	60	VLNT	inguinal	42,5	3/4
Phillips et al, Sep 2019	Prospective case serie	37	0%	100%	0	100%	Not specified	NR	sLVA	NA	6,5	4/4
Montag et al, 2019	Prospective cohort	24	0%	100%	0	100%	ISL stage I - III	43,6	VLNT	inguinal	18,0	5/5
Phillips et al, Jul 2019	Prospective case serie	29	0%	100%	100%	0%	Not specified	NR	sLVA	NA	8,5	4/4
Maruccia et al, Jun 2019	Retrospective cohort	21	0%	100%	0	100%	ISL stage II	25,3	VLNT	inguinal/omentum	32,0	5/5
Winters et al, 2019	Retrospective case serie	12	0%	100%	0	100%	Campisi stage 1 - 2a	93,6	sLVA	NA	12,0	3/4
Visconti et al, 2019	Prospective case serie	10	0%	100%	100%	0%	ISL stage II	64,7	VLNT	supraclavicular	12,0	4/4
Yasunaga et al, 2019	Retrospective case serie	30	7%	93%	100%	0%	ISL stage I - II	NR	sLVA	NA	12,2	4/4
Aljaaly et al, 2019	Retrospective cohort	15	0%	100%	0	100%	Cheng's stage II - IV	28,9	VLNT	submental	12,0	4/5
Aljindan et al, 2019	Retrospective cohort	58	26%	74%	66%	34%	Cheng's stage I - II	42,8	sLVA	NA	16,5	5/5
Hara et al, Nov. 2019	Retrospective case serie	56	11%	89%	NR	NR	Not specified	NR	sLVA	NA	14,5	3/4
Hara et al, Feb. 2019	Prospective cohort	109	6%	94%	95%	5%	ISL stage I - III	89,2	sLVA	NA	9,1	4/5
Johnson et al, 2019	Retrospective case serie	7	0%	100%	0%	100%	ISL Stage II	NR	VLNT	omentum	7,8	4/4
Ho et al, 2019	Retrospective cohort	76	NA	NA	61%	39%	Cheng's stage II - IV	60,9	VLNT	not reported	50,1	5/5
Leppäpuska et al, 2019	Retrospective cohort	27	0%	100%	0%	100%	Not specified	NR	VLNT	inguinal	9,9	4/5
Maruccia et al, Nov 2019	Retrospective case serie	16	19%	81%	100%	0%	ISL stage II - III	27,2	VLNT	omentum	26,3	4/4
Seki et al, 2019	Retrospective cohort	30	0%	100%	0%	100%	ISL stage II	28,0	sLVA	NA	NR	3/5
Hadiwattage et al, 2019	Prospective case serie	8	100%	0%	100%	0%	ISL stage II - III	NA	sLVA	NA	18,2	3/4
Suzuki et al, 2019	Retrospective case serie	36	8%	86%	100%	0%	Not specified	51,6	sLVA	NA	6,0	3/4
Chung et al, 2019	Retrospective case serie	18	0%	100%	56%	44%	Campisi stages 2-3-4	48,0	sLVA	NA	NR	2/4
Khan et al, 2019	Prospective case serie	27	0%	100%	0%	100%	Not specified	42	sLVA	NA	12,0	2/4
Pereira et al, 2018	Retrospective case serie	33	34%	66%	79%	21%	Not specified	113,4	sLVA	NA	19,2	4/4
Salgarello et al, 2018	Retrospective case serie	70	7%	93%	37%	63%	Not specified	56	LVA (not specified)	NA	NR	2/4
Visconti et al, 2018	Retrospective case serie	128	8%	92%	71%	29%	ISL stage I - II	64,7	sLVA	NA	NR	4/4
Liu et al, 2018	Prospective case serie	30	0%	100%	0%	100%	ISL stage I - II	72	VLNT	inguinal	22,1	4/4
Kenworthy et al, 2018	Retrospective cohort	38	3%	97%	16%	84%	Not specified	38,5	VLNT	omentum	NR	4/5
Agko et al, 2018	Prospective case serie	12	0%	100%	50%	50%	Not specified	32	VLNT	omentum	6,8	4/4
Hayashi et al, 2018	Retrospective cohort	55	0%	100%	100%	0%	ISL stage II	70,8	sLVA	NA	12,0	5/5
Akita et al, 2018	Retrospective cohort	45	NR	NR	100%	0%	ISL stage II - III	NR	VLNT	inguinal/supraclavicular/lateral thoracic/submental	6,0	3/5
Engel et al, 2018	Retrospective cohort	72	0%	100%	0%	100%	Cheng's stage I - IV	31,3	VLNT/sLVA	inguinal	33,3	5/5
Asuncion et al, 2018	Retrospective case serie	15	NR	NR	NR	NR	Not specified	NR	VLNT	submental	12,0	2/4
Seki et al, 2018	Prospective case serie	10	0%	100%	100%	0%	ILS stage II	69,1	sLVA	NA	7,7	3/4
Ho et al, 2018	Retrospective cohort	43	0%	100%	0%	100%	Not specified	46,1	VLNT	inguinal/submental	39,8	5/5

Gustafsson et al, 2018	Retrospective case serie	35	0%	100%	100%	0%	Cheng's stage II - IV	52,8	VLNT	submental	30,3	4/4
Ciudad et al, Oct 2017	Retrospective case serie	7	0%	100%	43%	57%	ISL stage III	29,1	VLNT	omentum	9,7	3/4
Arrivé et al, 2017	Retrospective case serie	15	0%	100%	0%	100%	Not specified	96	VLNT	inguinal	NA	4/4
Akita et al, 2017	Retrospective cohort	27	0%	100%	0%	100%	Not specified	NR	VLNT	inguinal	18,8	5/5
Gentileschi et al, 2017	Prospective case serie	16	0%	100%	0%	100%	ISL stage II	68,4	sLVA	NA	12,1	3/4
Winters et al, 2017	Retrospective case serie	29	0%	100%	0%	100%	Campisi stage 1b-2a	108	sLVA	NA	12,0	4/4
Gennaro et al, 2017	Retrospective case serie	37	11%	89%	73%	27%	ISl stage I - III	NR	sLVA	NA	NR	2/4
Poumellec et al, 2017	Retrospective case serie	31	0%	100%	0%	100%	Campisi stage 1 - 4	NR	sLVA	NA	12,7	4/4
Smith et al, 2017	Retrospective case serie	17	6%	76%	100%	0%	Not specified	156	VLNT	axillary	11,0	4/4
Inbal et al, 2017	Prospective case serie	11	0%	100%	27%	73%	ISL stage I - II	93	VLNT/LVA (not specified)	lateral thoracic	6,7	4/4
Gratzon et al, 2017	Prospective case serie	50	0%	100%	0%	100%	ISL stage I - II	58,8	VLNT	inguinal/lateral thoracic/supraclavicular	12,0	4/4
Nguyen et al, 2017	Prospective case serie	42	5%	88%	55%	45%	ICG-LDB stage 3 - 5	69,6	VLNT/sLVA	omentum	14,0	4/4
Cornelissen et al, 2017	Prospective cohort	20	0%	100%	0%	100%	ISL stage I - II	72	sLVA	NA	7,8	4/5
Coriddi et al, 2017	Retrospective case serie	15	0%	100%	46%	54%	Not specified	117,1	VLNT/sLVA	omentum	9,0	3/4
Ciudad et al, Mar 2017	Prospective case serie	10	0%	100%	50%	50%	ISL stage II-III	35,2	VLNT	omentum	14,7	4/4
De Brucker et al, 2016	Retrospective case serie	25	0%	100%	0%	100%	ISL stage I - II	47,8	VLNT	inguinal	29,0	3/4
Dionyssiou et al, 2016	Randomized controlled trial	18	0%	100%	0%	100%	ISL stage II	NA	VLNT	inguinal	18,0	Some concerns
Gennaro et al, 2016	Retrospective case serie	69	13%	87%	39%	61%	ISL stage III - IV (according to authors)	69,6	sLVA	NA	12,0	3/4
Chen et al, 2016	Retrospective case serie	21	19%	81%	38%	62%	Campisi stage I - IV	NR	VLNT/s- and mLVA	inguinal/supraclavicular	NR	2/4
Shih et al, 2016	Prospective case serie	5	20%	80%	40%	60%	ISL stage II	NR	sLVA	NA	9,0	3/4
Ito et al, 2016	Prospective case serie	5	40%	60%	100%	0%	ISl stage I - II	81,6	sLVA	NA	10,0	3/4
Masia et al, 2016	Prospective case serie	106	0%	100%	0%	100%	Not specified	NR	VLNT/sLVA	inguinal	18,0	4/4
Mihara et al, 2016	Retrospective case serie	84	18%	82%	100%	0%	ISl stage I - III	NR	sLVA	NA	18,3	3/4
Patel et al, Jul. 2015	Prospective cohort	25	7%	93%	40%	60%	Custom stage	60,5	VLNT	inguinal/submental	21,6	5/5
Yamamoto et al, Feb 2015	Retrospective cohort	14	0%	100%	100%	0%	ICG-LDB stage 2 - 4	NR	sLVA	NA	6,0	4/5
Seki et al, 2015	Retrospective cohort	30	0%	100%	100%	0%	ICG-LDB stage 1 - 4	52,4	sLVA	NA	12,0	5/5
Yamamoto et al, Oct 2015	Retrospective case serie	5	0%	100%	100%	0%	ICG-LDB stage 1 - 4	NR	sLVA	NA	6,0	3/4
Chen et al, 2015	Retrospective case serie	9	11%	89%	33%	77%	Campisi stage II - IV	NR	mLVA	NA	7,6	4/4
Akita et al, 2015	Retrospective cohort	56	20%	80%	100%	0%	Not specified	NR	VLNT/sLVA	thoracic/inguinal/supraclavicular	18,3	5/5
Nguyen et al, 2015	Retrospective case serie	29	0%	100%	0%	100%	Not specified	39,6	VLNT	inguinal	11,0	4/4
Mihara et al, 2014	Retrospective case serie	95	7%	93%	88%	12%	ISL stage 0 - III	NR	sLVA	NA	27,3	4/4
Chen et al, 2014	Retrospective case serie	10	0%	100%	0%	100%	Not specified	NR	VLNT	inguinal	NR	3/4
Ayestaray et al, 2014	Prospective case serie	20	NR	NR	40%	60%	Campisi staging (mean 3.3)	74,4	sLVA	NA	NR	3/4
Granzow et al, 2014	Prospective cohort	16	25%	75%	44%	56%	Not specified	72,6	VLNT/sLVA	inguinal	29,5	5/5
Yamamoto et al, Jan 2014	Prospective cohort	12	0%	100%	100%	0%	ICG-LDB stage 2-5	NR	sLVA	NA	NR	3/5
Yamamoto et al, Jul 2014	Prospective cohort	6	0%	100%	100%	0%	ISL stage I	NR	sLVA	NA	6,0	3/5
Yamamoto et al, Jan 2014	Prospective cohort	11	NR	NR	73%	27%	ICG-LDB stage 2-3	NR	sLVA	NA	6,0	3/5
Yamamoto et al, Feb 2014	Prospective cohort	8	0%	100%	63%	38%	ICG-LDB stage 2 -5	NR	sLVA	NA	6,0	3/5
Patel et al, Jan 2015	Retrospective case serie	20	0%	100%	35%	65%	Not specified	51,2	VLNT	inguinal/submental	27,3	4/4
Chang et al, 2013	Prospective cohort	100	0%	100%	11%	89%	Not specified	45,6	sLVA	NA	29,1	5/5
Akita et al, 2013	Prospective cohort	53	NR	NR	100%	0%	Not specified	NR	sLVA	NA	NR	4/5
Cheng et al, 2013	Prospective cohort	10	0%	100%	0%	100%	ISL stage II	33,2	VLNT	inguinal	36,6	5/5
Viitanen et al, 2013	Prospective cohort	14	0%	100%	0%	100%	Not specified	54,2	VLNT	inguinal	33,5	5/5
Boccardo et al, 2013	Retrospective cohort	41	0%	100%	100%	0%	ISL stage I - II	NR	mLVA	NA	42,0	3/4
Ayestaray et al, 2013	Prospective case serie	20	NR	NR	0%	100%	Campisi stage II - V	38,4	sLVA	NA	NR	3/4
Yamamoto et al, 2013	Prospective cohort	14	NR	NR	100%	0%	ISL stage I - II	NR	sLVA	NA	6,0	3/5
Mihara et al, 2012	Retrospective case serie	6	0%	100%	100%	0%	ISL stage 0 - I	10	sLVA	NA	6,0	3/4
Auba et al, 2012	Retrospective cohort	10	0%	92%	50%	50%	Campisi stage II - V	137	sLVA	NA	NR	3/5
Saaristo et al, 2012	Prospective cohort	9	0%	100%	0%	100%	Not specified	43	VLNT	inguinal	NR	4/5
Maegawa et al, Mar 2012	Retrospective cohort	57	11%	89%	100%	0%	ISL stage I - III	NR	sLVA	NA	14,0	5/5
Cheng et al, 2012	Prospective case serie	6	0%	100%	100%	0%	ISL stage II - III	71	VLNT	submental	8,7	3/4
Maegawa et al, May 2012	Retrospective cohort	31	0%	100%	100%	0%	ISL stage I - III	NR	sLVA	NA	17,0	5/5
Lee et al, 2011	Prospective cohort	32	31%	69%	90%	10%	Custom stage	NR	VLNT/s- and mLVA	posterior axillary	48,0	5/5
Furukawa et al, 2011	Prospective case serie	9	0%	100%	0%	100%	Not specified	87,6	mLVA	NA	17,0	3/4
Mihara et al, 2011	Prospective case serie	11	18%	82%	100%	0%	ISL stage I - II	24,2	LVA (not specified)	NA	23,6	3/4
Gharb et al, 2011	Prospective cohort	21	0%	100%	0%	100%	ISL stage II	NR	VLNT	inguinal	43,0	5/5
Chang et al, 2010	Prospective case serie	20	0%	100%	0%	100%	Campisi stage II - III	57,6	sLVA	NA	12,0	3/4
Narushima et al, 2010	Prospective cohort	14	0%	100%	86%	14%	Campisi stage II - IV	86,4	sLVA	NA	8,9	4/5
Demirtas et al, 2010	Prospective cohort	78	77%	23%	100%	0%	Campisi stage (mean 2.8)	86,9	s- and mLVA	NA	NR	4/5
Demirtas et al, 2009	Prospective cohort	42	81%	19%	100%	0%	Campisi stage II - IV	93,6	s- and mLVA	NA	11,8	5/5
Damstra et al, 2009	Prospective case serie	10	0%	100%	0%	100%	Campisi stage III	63,6	sLVA	NA	NR	3/4
Lin et al, 2009	Prospective case serie	13	0%	100%	0%	100%	Not specified	32,2	VLNT	inguinal	56,3	3/4
Becker et al, 2006	Retrospective case serie	24	0%	100%	0%	100%	Custom stage	51,7	VLNT	inguinal	99,6	3/4
Matsubara et al, 2006	Retrospective case serie	9	0%	100%	100%	0%	ISL stage II	136,8	sLVA	NA	68,0	3/4
Koshima et al, 2004	Retrospective case serie	52	NR	NR	100%	0%	Campisi stage III - IV	63,6	sLVA	NA	14,5	3/4
Koshima et al, 2003	Prospective cohort	13	24%	76%	100%	0%	Not specified	80,4	sLVA	NA	55,2	5/5
Yamamoto et al, 2003	Retrospective case serie	18	0%	100%	0%	100%	Custom stage	85,2	mLVA	NA	24,0	3/4
Koshima et al, 2000	Retrospective cohort	12	4%	92%	0%	100%	Custom stage	98,4	sLVA	NA	26,4	5/5
Koshima et al, 1996	Prospective case serie	14	14%	86%	57%	43%	Not specified	95,8	LVA (not specified)	NA	24,3	3/4
Campisi et al, 1995	Retrospective case serie	664	18%	81%	90%	10%	Not specified	NR	sLVA	NA	60,0	2/4
O'Brien et al, 1990	Retrospective case serie	52	0%	100%	12%	88%	Not specified	NR	sLVA	NA	42,6	5/5
