# Supplementary material for: Outcomes after microsurgical treatment of lymphedema: a systematic review and meta-analysis
Source: Int J Surg. 2023 Apr 17;109(5):1360–72. doi: 10.1097/JS9.0000000000000210 (PMC10389392; doi:10.1097/JS9.0000000000000210)
Supplement: Supplementary file 5 [file js9-109-1360-s005.pdf]

Search strategies

Medline All

(exp Breast Cancer Lymphedema/ OR exp Non-Filarial Lymphedema/ OR Lymphedema/ OR (lymphedema OR lymphoedema).ab,ti,kf.) AND (Microsurgery/ OR Anastomosis, Surgical/ OR Lymph Nodes/tr OR Lymphatic Vessels/su OR (microsurger* OR micro-surger* OR (surger* ADJ3 (microvascular OR microscale OR microscopic)) OR bypass* OR micro#anastomos* OR anastomos* OR (lymph* ADJ3 (transfer* OR graft*))).ab,ti,kf.) NOT (exp animals/ NOT humans.sh.)

1265	**References found**	**31 janvier 2022**

Embase.com

('lymphedema'/exp OR lymph*dema:ab,ti,kw) AND ('microsurgery'/de OR 'microvascular surgery'/exp OR 'anastomosis'/de OR 'bypass'/de OR 'end to end anastomosis'/de OR 'end to side anastomosis'/exp OR 'side to side anastomosis'/exp OR (microsurger* OR micro-surger* OR (surger* NEAR/3 (microvascular OR microscale OR microscopic)) OR bypass* OR micro$anastomos* OR anastomos* OR (lymph* NEAR/3 (transfer* OR graft*))):ab,ti,kw) NOT ('animal'/exp NOT 'human'/exp)

1536	**références trouvées le**	**31 janvier 2022**

PubMed

(lymphedema[tiab] OR lymphoedema[tiab]) AND (((microvascular[tiab] OR microscale[tiab] OR microscopic[tiab]) AND surger*[tiab]) OR microsurger*[tiab] OR micro-surger*[tiab] OR anastomos*[tiab] OR microanastomos*[tiab] OR micro-anastomos*[tiab] OR bypass*[tiab] OR (lymph*[tiab] AND (transfer*[tiab] OR graft*[tiab]))) NOT medline[sb]

232	**références trouvées le**	**31 janvier 2022**

CINAHL with Full Text EBSCO

(MH "Lymphedema" OR TI(lymph*dema) OR AB(lymph*dema)) AND (MH "Anastomosis, Surgical" OR MH "Microsurgery" OR MH "Lymph Nodes/TR" OR TI(microsurger* OR micro-surger* OR (surger* N3 (microvascular OR microscale OR microscopic)) OR bypass* OR micro#anastomos* OR anastomos* OR (lymph* N3 (transfer* OR graft*))) OR AB(microsurger* OR micro-surger* OR (surger* N3 (microvascular OR microscale OR microscopic)) OR bypass* OR micro#anastomos* OR anastomos* OR (lymph* N3 (transfer* OR graft*))))

97	**références trouvées le**	**1er février 2022**

Cochrane Central Register of Controlled Trials – Wiley

lymph*dema:ab,ti,kw AND (microsurger* OR micro-surger* OR (surger* NEAR/3 (microvascular OR microscale OR microscopic)) OR bypass* OR micro?anastomos* OR anastomos* OR (lymph* NEAR/3 (transfer* OR graft*))):ab,ti,kw

55	**références trouvées le**	**1er février 2022**

ProQuest Dissertations & Theses A&I

TI,AB((lymph*dema) AND (microsurger* OR micro-surger* OR (surger* NEAR/3 (microvascular OR microscale OR microscopic)) OR bypass* OR micro?anastomos* OR anastomos* OR (lymph* NEAR/3 (transfer* OR graft*))))

3	**références trouvées le**	**1er février 2022**
