# Supplementary material for: Outcomes after microsurgical treatment of lymphedema: a systematic review and meta-analysis
Source: Int J Surg. 2023 Apr 17;109(5):1360–72. doi: 10.1097/JS9.0000000000000210 (PMC10389392; doi:10.1097/JS9.0000000000000210)
Supplement: Supplementary file 6 [file js9-109-1360-s006.pdf]

Review title or ID	
Study ID *(surname of first author and year first full report of study was published e.g. Smith 2001)*	
PMID	
Associated PMID (errata, comments or retractions)	
Notes	

General Information

Date form completed *(dd/mm/yyyy)*	
Name/ID of person extracting data	
Reference citation	
Study author contact details	
Publication type *(e.g. full report, abstract, letter)*	
Notes:	

Study eligibility

Study Characteristics	Eligibility criteria	Eligibility criteria met? Yes	No	Unclear
Type of study	Randomised Controlled Trial	☐	☐	☐
	Prospective or retrospective cohort	☐	☐	☐
	Prospective or retrospective case-serie (with or without controls)	☐	☐	☐
	Other design requiring closer assessment :	☐	☐	☐
Participants	Adults > 18 y.o. with primary or secondary lymphedema (not parasitic) with documented characteristics	☐	☐	☐
Types of intervention	LVA or VLNT (including combination with flap reconstruction	☐	☐	☐
Types of comparison	Any	☐	☐	☐
Outcomes:	Volume	☐	☐	☐

	Circumference	☐	☐	☐
	Number of compressive decongestive therapy (CDT) per defined period of time	☐	☐	☐
	Use of compressive garments	☐	☐	☐
	Number of infectious episodes per defined period of time	☐	☐	☐
	Lymphoscintigraphy	☐	☐	☐
	Lymphography	☐	☐	☐
	MRI/CT/SPECT-CT	☐	☐	☐
	Quality of life (QoL)	☐	☐	☐
	Patient satisfaction	☐	☐	☐

INCLUDE ☐	
Reason for exclusion	

DO NOT PROCEED IF STUDY EXCLUDED FROM REVIEW

Characteristics of included studies

Methods

	**Descriptions as stated in report/paper**
**Design**	
**Start date**	
**End date**	

Participants

	Description<br>*Include comparative information for each intervention or comparison group if available*	Location in text or source *(pg & ¶/fig/table/other)*
Population description		
Inclusion criteria		
Exclusion criteria		
Total no. of patients included		

Withdrawals and exclusions		
Age + SD		
Sex		
BMI + SD		
Etiology of lymphedema		
Location of lymphedema		
Preop. conservative therapy (Y/N)		
Stages of lymphedema (prefer ISL)		
Duration of lymphedema (since diagnosis)		

Intervention

	Description as stated in report/paper
Type of intervention	
Co-interventions	
Number of anastomosis performed if LVA	

Outcomes

NB: Duplicate table if several outcomes reported

	Description as stated in report/paper
Outcome :	
Unit and technique of measurement	

Risk of Bias assessment

Refer to NOS form and Cochrane risk of bias form

Data and analysis.

	Description as stated in report/paper					
Outcome						
Time point/follow-up						
Results	Intervention			Comparison (if applicable)		
	Mean	SD *(or other variance, specify)*	No. participants	Mean	SD *(or other variance, specify)*	No. participants
Any other results reported *(e.g. mean difference, CI, P value)*						
Statistical methods used and appropriateness of these *(e.g. adjustment for correlation)*						
Reanalysis required? *(specify)*	☐ Yes ☐ No ☐ Unclear					
Reanalysis possible?	☐ Yes ☐ No ☐ Unclear					
Reanalysed results						

Other information

	**Description as stated in report/paper**
**Key conclusions of study authors**	
**References to other relevant studies**	
**Need to contact authors?**	
